# Altered Expression of *OsAAP3* Influences Rice Lesion Mimic and Leaf Senescence by Regulating Arginine Transport and Nitric Oxide Pathway

**DOI:** 10.3390/ijms22042181

**Published:** 2021-02-22

**Authors:** Qilang Wei, Zhenwei Yan, Yifan Xiong, Zhongming Fang

**Affiliations:** 1 Key Laboratory of Plant Resource Conservation and Germplasm Innovation in Mountainous Region (Ministry of Education), College of Agricultural Sciences, Guizhou University, Guiyang 550025, China; weiqilang9@gmail.com; 2 Xiamen Plant Genetics Key Laboratory, School of Life Sciences, Xiamen University, Xiamen 361102, China; 201890900012@sdu.edu.cn; 3 Hubei Engineering Research Center of Viral Vector, Wuhan University of Bioengineering, Wuhan 430415, China; ewanxiong@gmail.com

**Keywords:** rice, lesion mimic, leaf senescence, arginine transport, nitric oxide

## Abstract

Persistent lesion mimic can cause leaf senescence, affecting grain yield in crops. However, knowledge about the regulation of lesion mimic and leaf senescence in crop plants is still limited. Here, we report that the amino acid transporter OsAAP3, a negative regulator of tiller bud elongation and rice grain yield, is involved in lesion mimic and leaf senescence. Altered expression of *OsAAP3* can initiate the nitric oxide signaling pathway through excessive accumulation of arginine in rice leaves, influencing ROS accumulation, antioxidant enzymes activities, proline concentration, and malondialdehyde concentration. This finally triggers cell death which ultimately leads to lesion mimic and leaf senescence by regulating the degradation of chloroplast and the expression abundance of components in the photosynthetic pathway. Overall, the results not only provide initial insights into the regulatory role of amino acid transport genes in rice growth and development, but also help to understand the factors regulating the leaf senescence.

## 1. Introduction

Lesion mimics refer to the disease spots spontaneously produced on plant leaves, stems, and leaf sheaths without pathogen infection or environmental stress. The formation of lesion mimics is closely related to cell development and plant defense. Recently, some lesion mimic-related genes have been found in rice to control cell death in the defense response of pathogens. For example, a loss of *SPL33* function accelerated leaf senescence caused by H_2_O_2_ accumulation [1]. Furthermore, mutant *spl29* has been shown to affect leaf lesions and senescence in rice [2]. Moreover, *SPL35* plays an important role in rice cell death [3]. Importantly, if lesion mimics persist, the drastic changes of leaf metabolism will lead to the degradation of metabolites and the mobilization of nutrients to developing tissues and organs, and eventually result in leaf senescence in rice [4]. 

Reactive oxygen species (ROS) are thought to play a vital role in plant senescence [5,6]. In previous studies, higher levels of ROS in cells caused serious oxidative damage to membranes, nucleic acids and proteins [7,8]. Furthermore, accumulation of unnecessary ROS induces leaf senescence and may even be related to programmed cell death (PCD). So far, it has been reported that the premature leaf senescence 1 (*ospls1*) mutant in rice accumulates excess ROS, resulting in a phenotype of early leaf senescence [9]. The rice *SPL4* gene also plays an important role in leaf senescence through inhibited ROS accumulation in leaf development [10]. Recently, mutation in a putative glycosyltransferase like gene causes programmed cell death and early leaf senescence in rice [11]. In addition, ROS are regulated by nitric oxide (NO), which can directly affect the activity of antioxidant enzymes such as superoxide dismutase (SOD), CAT (catalase), and POD (Peroxidase) and increase the content of H_2_O_2_ in plants, thereby starting and accelerating the process of ROS regulation [12]. 

Importantly, NO is a key regulator of leaf senescence and PCD in higher plants, which regulates a variety of physiological processes [13,14]. Furthermore, arginine (Arg) is a precursor of NO in plants and produces NO in a reaction catalyzed by nitric oxide synthase (NOS) [15]. In rice, Arg can be transported by amino acid transporter OsAAP3 [16]. Recently, we found that down regulating the expression of *OsAAP3* promoted tiller number and grain yield by increasing outgrowth buds in rice [17]. Furthermore, *OsAAP3* overexpressing (OE) lines accumulated too much Arg and lysine (Lys), which inhibited the elongation of tiller buds as well as the number of tillers, and decreased the grain yield of rice [17]. Interestingly, *OsAAP3* OE lines showed lesion mimics followed by leaf senescence during the reproductive period, and the underlying mechanism is still not clear. The objective of the present study was to investigate the roles of *OsAAP3* in lesion mimic and leaf senescence by regulating Arg transport and nitric oxide pathway. 

## 2. Results

### 2.1. Over-Expression of OsAAP3 Leads to Lesion Mimic and Leaf Senescence in Rice

**Figure 1:**
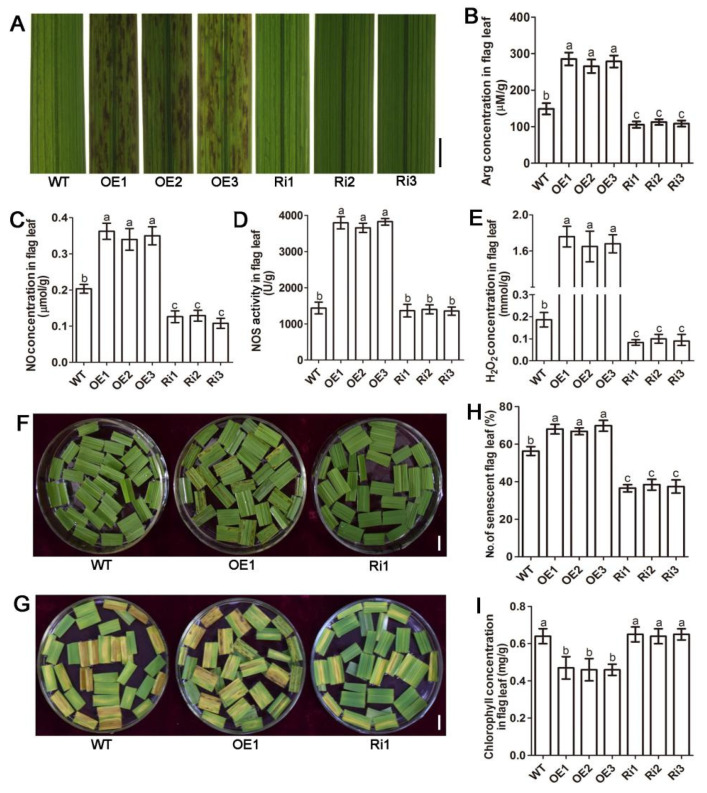
*OsAAP3* leads to lesion mimic and leaf senescence in rice. Flag leaf phenotype (**A**), arginine (Arg) concentration (**B**), nitric oxide (NO) concentration (**C**), nitric oxide synthase (NOS) activity (**D**), and hydrogen peroxide (H_2_O_2_) concentration (**E**) of the *OsAAP3* overexpressing (OE) lines, RNA interference (Ri) lines, and wild-type (WT) at the heading stage. Flag leaf phenotype of the *OsAAP3* OE lines, Ri lines, and WT with H_2_O (**F**) and H_2_O_2_ (**G**) treatments for 4 d. The number of senescent yellow leaves (**H**) and chlorophyll concentration (**I**) for flag leaves of the *OsAAP3* OE lines, Ri lines and WT with H_2_O_2_ treatment for 4 d. The flag leaves was taken at 110 days after sowing. Scale bar = 0.5 cm in A, F, G. The letters above the error bars are ranked by the Duncan test at *p* < 0.05. Values are means ± standard deviation (*n* = 5).

Our previous study reported that blocking *OsAAP3* could promote rice tillering and grain yield by regulating tiller bud elongation [17]. Surprisingly, over-expression of *OsAAP3* not only decreases the number of tillers and grain yield of rice [17], but also leads to lesion mimic and leaf senescence in rice, especially in flag leaves (Figure 1A, Supplementary Materials Figure S1). To dissect the underlying mechanism of this process, we detected the Arg concentrations in the flag leaves of *OsAAP3* OE lines, RNA interference (Ri) lines, and wild-type (WT). The results showed that the concentration of Arg in the flag leaf of the OE lines was significantly higher than that in the WT, while the Arg concentration in the Ri lines was significantly decreased compared with that in the WT (Figure 1B), proving that an increase in *OsAAP3* expression could directly lead to the excessive accumulation of Arg in rice leaves.

Arg is not only an important nitrogen storage nutrient for reuse but also a precursor of NO in plants [18], and NO is an important signal molecule in plants [19]. Therefore, we measured the concentration of NO in the leaves of all materials. The results showed that the concentration of NO in the OE lines was significantly higher than that in the WT, while the NO concentration in the Ri lines was significantly lower than that in the WT (Figure 1C). Meanwhile, we also measured the activity of NOS, and found that the NOS activity in the OE lines was significantly higher than that in the WT, but there was no significant change in the activity of NOS between the Ri lines and WT (Figure 1D).

It was further found that the concentration of H_2_O_2_ in the OE lines was significantly higher than that in the WT, while the concentration in the Ri lines was lower than that in the WT (Figure 1E). Moreover, to further determine the effect of H_2_O_2_ concentration on leaf senescence, flag leaves from the OE lines, Ri lines, and WT at the same growth stage were soaked in vitro with H_2_O (Figure 1F) and H_2_O_2_ (Figure 1G) for 4 d. The results showed that the number of senescent yellow leaves in the OE lines was significantly greater than that in the WT; however, there were obviously fewer senescent yellow leaves in the Ri lines compared to those in the WT (Figure 1H). In addition, the chlorophyll concentration in the OE lines was significantly lower than that in the WT, but the chlorophyll concentration of the Ri lines was similar to that of the WT, confirming that leaf senescence in the OE lines may be related to chloroplast degradation (Figure 1I).

### 2.2. OsAAP3 Regulates Leaf-Senescence Induced by Reactive Oxygen Species

To further understand the physiological mechanism of leaf senescence regulated by *OsAAP3*, 3,3′-diaminobenzidine (DAB), nitro blue tetrazolium (NBT), and trypan blue (TB) were used to treat rice flag leaves from the OE lines, Ri lines, and WT. The results showed that there were more staining sites for the OE lines than for the WT with DAB and NBT staining, while there were fewer staining sites for the Ri lines than for the WT (Figure S2A,B), indicating that both the hydrogen peroxide and superoxide in the flag leaves of the OE lines is higher than that in the WT. In accordance, the result from the TB dyeing suggested that rice leaf premature aging in the OE lines is most likely caused by the accumulation of ROS (Figure S2C).

**Figure 2:**
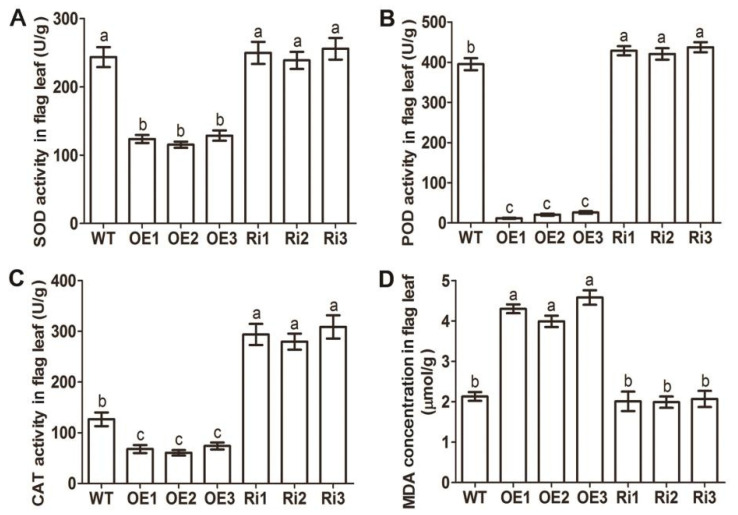
Superoxide dismutase (SOD) activity (**A**), peroxidase (POD) activity (**B**), catalase (CAT) activity (**C**), and malondialdehyde (MDA) concentration (**D**) of the *OsAAP3* OE lines, Ri lines, and wild-type (WT) at the heading stage. The flag leaves was taken at 110 days after sowing. The letters above the error bars are ranked by the Duncan test at *p* < 0.05. Values are means ± standard deviation (*n* = 5).

Furthermore, the SOD activity, POD activity, CAT activity, proline (Pro) concentration, and malondialdehyde (MDA) concentration were analyzed in the flag leaves of *OsAAP3* transgenic materials. As shown in Figure 2, the SOD (Figure 2A), POD (Figure 2B), and CAT activities (Figure 2C) in the OE lines were significantly lower than those in the WT, while the POD (Figure 2B) and CAT activities (Figure 2C) in the Ri lines were significantly higher than those in the WT. These results indicated that the ability to remove H_2_O_2_ and oxygen ions in the flag leaves of OE lines was significantly weaker than that in the WT and Ri lines. Conversely, the Pro (Figure S3) and MDA concentrations (Figure 2D) were significantly higher in the OE lines than those in the WT, while the Pro concentration was significantly lower in the Ri lines than that in the WT (Figure S3).

### 2.3. The Leaf Senescence Regulated by OsAAP3 Is Related to Chloroplast Degradation and Cell Death

**Figure 3:**
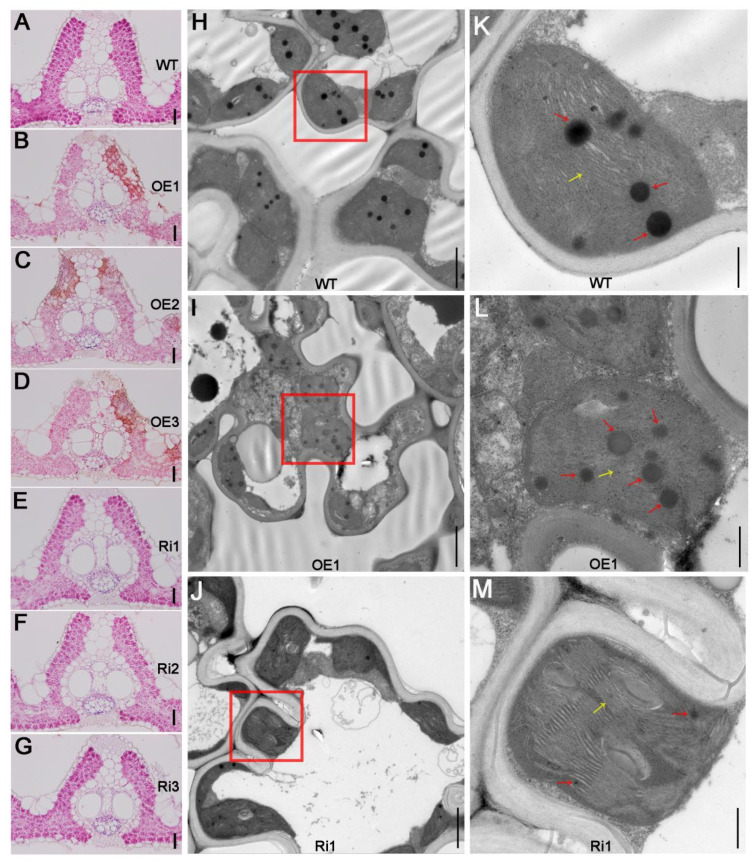
Leaf senescence regulated by *OsAAP3* is related to chloroplast degradation and cell death. Paraffin sections were used to detect the chloroplasts in the wild-type (WT) (**A**), overexpressing (OE) lines (**B**–**D**), and RNA interference (Ri) lines (**E**–**G**). Transmission electron sections were used to detect the chloroplasts in the WT (**H**,**K**), OE lines (**I**,**L**), and Ri lines (**J**,**M**). Paraffin sections (**A**–**G**) were stained with hematoxylin for 5 min and eosin for 2 min.The red frames in (**H**–**J**) indicate chloroplasts, the yellow arrows in (**K**–**M**) indicate thylakoids, and the red arrows in (**K**–**M**) indicate plastoglobules. The flag leaves was taken at 110 days after sowing. Scale bar = 30.0 mm in (**A**–**G**), 2.0 μm in (**H**–**J**), and 0.5 μm in (**K**–**M**).

To determine what kind of changes occur in the inner tissues of leaves due to leaf senescence in *OsAAP3* OE lines, paraffin sections and transmission electron microscopy sections were used to observe the leaves. Histochemical staining of rice flag leaves paraffin sections showed that the nuclei of WT and Ri lines were stained blue by hematoxylin, while the nuclei of cells within abnormal staining by eosin of OE lines were not stained blue, indicating that the *OsAAP3* OE lines presented cell death in leaf mesenchymal cells with a typical cell structure of dell death, and the Ri lines and WT showed no obvious abnormal cell death (Figure 3A–G). Further observation of the flag leaf tissues transmission electron microscopy showed that the chloroplast structure in the leaf mesenchymal cells began to degrade in the OE lines, the stromal lamella of thylakoid structure disintegrated, and the mitochondria also showed obvious vacuolation, which was more obvious than that in the WT plants (Figure 3H–L). However, in the mesophyll cells of the Ri transgenic plants, the chloroplast structure was intact, the thylakoid structure was clearly visible, and the number of osmiophilic particles was significantly reduced (Figure 3J–M). These results suggest that *OsAAP3* may affect leaf senescence by regulating the degradation of chloroplasts in the mesophyll cells of flag leaves.

### 2.4. Transcriptome Analysis Reveals That Over-Expression of OsAAP3 Leads to the Abnormal Gene Expression of Secondary Metabolism and Photosynthesis Pathway

**Figure 4:**
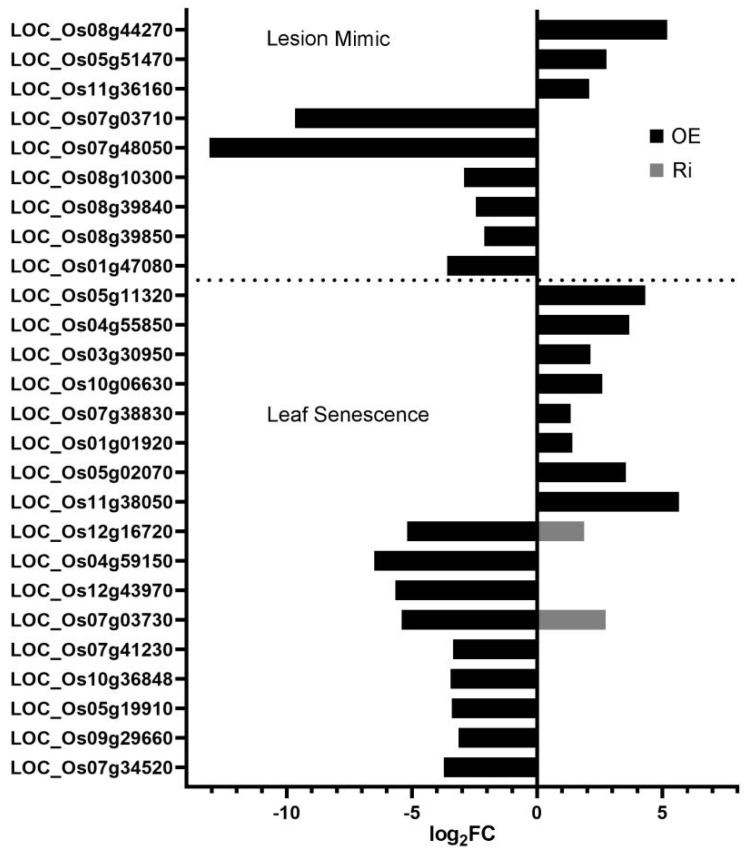
Differentially enriched genes in the lesion mimic and leaf senescence pathways. OE indicates the overexpression line, Ri indicates the RNA interference line, abscissa indicates a log2 fold change, and ordinate indicates the locus name of the gene.

**Figure 5:**
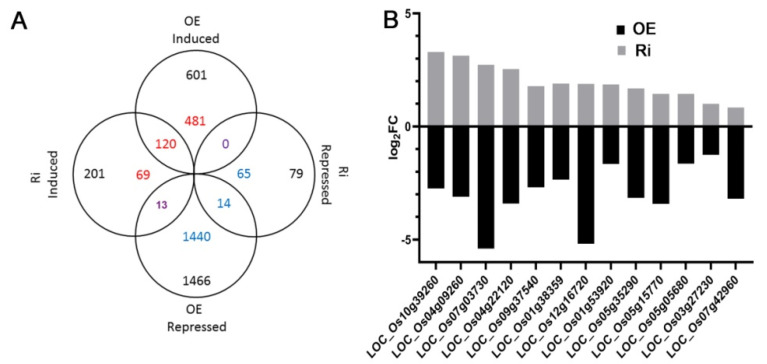
Differentially expressed genes among the OE line, Ri line, and WT. (**A**) Venn map of differential genes among the overexpressing (OE) line, RNA interference (Ri) line, and wild-type (WT). (**B**) 13 differentially expressed genes among the OE line, Ri line, and WT. Abscissa indicates a log2 fold change, and ordinate indicates the locus name of the genes.

To investigate the mechanism of *OsAAP3* in regulating lesion mimic and leaf senescence, we performed RNA-seq using RNA samples from the leaves of the OE lines, Ri lines, and WT. MAPMAN was used to annotate the differentially expressed genes in the OE line and Ri line leaves to obtain the distribution of differentially expressed genes in different metabolic pathways (Figure S4). The results showed that there were more differentially expressed genes in the photosynthesis, amino acid metabolism, and nitrogen metabolism pathways in both the OE and Ri lines compared with those in the WT (Figure S4). However, the OE lines (Figure S4A) had more differentially expressed genes in these pathways than those in the Ri lines (Figure S4B). Besides, the expression levels in the flavonoids pathway of OE and Ri lines changed compared with WT (Figure S4). And the expression levels in the phenolics pathway of OE lines decreased, but that were almost unaffected in Ri lines (Figure S4). Furthermore, we customized the figure to depict the biological processes of interest from MAPMAN metabolic pathways in Figure S4. The result showed that three genes were up-regulated and six genes were down-regulated in the lesion mimic pathway in the OE line leaves compared with those in the WT (Figure 4). Moreover, eight genes were up-regulated and nine genes were down-regulated in the OE line leaves in the leaf senescence pathway, while two genes in the Ri lines showed the opposite expression compared with that in the OE lines (Figure 4). In addition, the expression of 16 genes in the nitrogen metabolism pathway and 8 genes in the photosynthesis pathway were also changed in the OE and Ri lines compared with those in the WT (Figure S5); the expression levels of these genes were confirmed by real-time quantitative PCR (RT-PCR) (Figure S6). The Venn diagram shows the number of differentially expressed genes in the OE and Ri lines compared with that in the WT (Figure 5A). A total of 13 genes encoding for enzymes involved in nitrogen metabolism, chloroplast development and protein phosphorylation, have opposite expression trends in the OE and Ri lines (Figure 5B; Table S1). In addition, we found that 11 genes were highly expressed in the OE line, and 11 genes exhibited lower expression in the OE line by RT-PCR analysis (Figure S7), confirming that these genes might play important roles in the regulation of lesion mimic and leaf senescence. Furthermore, the similar expression trends of these genes between the RT-PCR results and the RNA-seq results illustrated the good quality of our transcriptomic data. The locus name and corresponding notes for these genes in all the above pathways are shown in Table S1.

### 2.5. Altered Expression of OsAAP3 Affects Nutrient Elements in Rice

**Figure 6:**
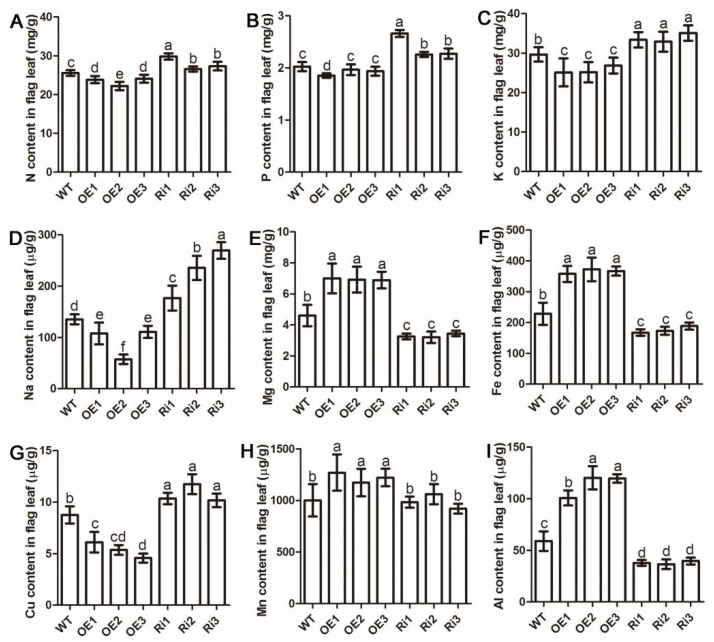
Measurements of the content of different nutrient elements in flag leaves among the OE lines, Ri lines, and WT. Nitrogen (N, **A**), phosphorus (P, **B**), potassium (K, **C**), sodium (Na, **D**), copper (Cu, **E**), magnesium (Mg, **F**), iron (Fe, **G**), aluminum (Al, **H**), and manganese (Mn, **I**) contents among the overexpressing (OE) lines, RNA interference (Ri) lines, and wild-type (WT). The flag leaves was taken at 110 days after sowing. The letters above the error bars are ranked by the Duncan test at *p* < 0.05. Values are means ± standard deviation (*n* = 5).

To further investigate the effect of *OsAAP3* on nutrient elements in rice, the element concentration in the flag leaves of different *OsAAP3* transgenic lines was further detected. The results showed that the concentrations of nitrogen (N, Figure 6A), phosphorus (P, Figure 6B), and potassium (K, Figure 6C), which are the most prominent elements necessary for the growth and development of rice, were higher in the Ri lines than those in the WT and OE lines. In addition, sodium (Na, Figure 6D), which is related to abiotic adversity, and copper (Cu, Figure 6E), which is related to biological adversity, also showed a similar trend. On the other hand, the accumulations of magnesium (Mg, Figure 6F), iron (Fe, Figure 6G), aluminum (Al, Figure 6H) and manganese (Mn, Figure 6I) in the OE lines were significantly higher than those in the WT and Ri lines. These results suggest that the altered expression of *OsAAP3* significantly changes the nutrient elements of rice flag leaves, which may be caused by the degradation of chloroplasts in the mesophyll cells.

## 3. Discussion

### 3.1. Over Expression of OsAAP3 Resulted in ROS Accumulation in Rice Leaves

In a previous study, we found that amino acid transporter gene *OsAAP3* was mainly expressed in root, leaf blade, leaf sheath, culm and panicle [17]. In addition, OsAAP3 mainly transports Lys and Arg from intercellular space to plant cells, and higher concentration of exogenous Arg inhibit the tiller bud elongation and seedling growth through the root uptake by OsAAP3 in rice [16,17]. Except for tillering, the effect of Arg transport by OsAAP3 on the development of other organs in rice has not been concerned. Excitingly, it was found that the accumulation of Arg in the leaves of the *OsAAP3* OE lines resulted in regulating of lesion mimic and leaf senescence (Figure 1A) by an increase in NO compared with that in the WT (Figure 1B,C). Significantly, the results showed that the concentration of H_2_O_2_ accumulated in the rice leaves of the *OsAAP3* OE lines at higher levels compared with that in the WT (Figure 1E).

SOD catalyzes the conversion of O_2_^−^ to H_2_O_2_, while POD and CAT catalyze the conversion of H_2_O_2_ to H_2_O in various organelles, and they provide a fence against oxidative stress [20]. These antioxidant enzymes play an important role in eliminating ROS to maintain normal plant metabolism [21] and are often involved in scavenging active oxygen [22]. We indicated that the activities of SOD, POD and CAT in the *OsAAP3* OE lines were decreased, while the POD and CAT activities increased in the *OsAAP3* Ri lines (Figure 2A–C), suggesting that the reduction of ROS-scavenging enzymes might result in an oxidative burst in the leaves of the *OsAAP3* OE lines. The decrease of the activities of these antioxidant enzymes may be due to the lower concentration of total free amino acids in the leaves of OE lines compared with that in WT [17]. Similarly, a previous study has shown that the activities of antioxidant enzymes were gradually lowered, leading to the aggravation of membrane lipid peroxidation with the proceeding N-deficiency [23]. In addition, proline levels increase proportionally with leaf age in excised leaf segments and are an indicator of leaf senescence [24,25]. In the present study, Pro accumulated in the *OsAAP3* OE lines, but decreased in the Ri lines (Figure S3), indicating that Pro may be involved in the ROS pathway. As the MDA content indirectly reflects the degree of cellular damage [26], our results also showed that the MDA content in the *OsAAP3* OE lines was significantly increased (Figure 2D), as observed for the *ell1* mutant with ROS accumulation and lesion formation [27].

### 3.2. Over Expression of OsAAP3 Triggers Cell Death and Lesion Mimic in Rice Leaves

In rice, ROS play a complex role as secondary messengers in the signaling pathways leading to PCD [28], and excessive ROS lead to oxidation of cell membrane, thereby influencing cell permeability, and eventually lead to plant cell death and leaf spot-like lesions [29,30]. Moreover, nuclear degradation and DNA fragmentation are important markers of cell death [11]. In the present study, we found that nuclei of part cells in the leaves of OE lines were not stained blue (Figure 3), suggesting that altered expression of *OsAAP3* could affect cell death in leaves. Besides, bursts of production of NO and ROS usually occur very early in the defense response and are often important for the initiation of hypersensitive response, together with lipid peroxidation, transcriptional reprogramming, ion fluxes and cell wall fortification [31]. We suggest that our findings about *OsAAP3* in cell death and lesion mimic in rice leaves are linked to the hypersensitive response, like some mutants display hypersensitive response-like lesion in the absence of pathogens attacks [11,32].

To further examine whether ROS are involved in cell death in the *OsAAP3* OE lines, NBT staining for superoxide accumulation, DAB staining for H_2_O_2_ accumulation, and TB staining for membrane damage were used for evaluation of the degree of ROS to cell death (Figure S2A–C). The staining results indicated that the *OsAAP3* OE lines might give rise to the production of cell death in rice leaf. In recent years, there are many reports about the mechanism of ROS mediated cell death [2,27,33]. For example, the mutation in *ELL1* disrupted the structure of chloroplast, then further caused accumulation of ROS, which ultimately triggered cell death of rice leaf [27]. Similarly, our results indicated that *OsAAP3* OE also may play an important role in ROS mediated cell death. However, over-expression of *OsAAP5* did not affect leaf development [34]. The possible reason is that the specific affinity of OsAAP5 for Arg transport is not as high as that of OsAAP3, and OsAAP5 also has strong transport activity for neutral amino acids [34]. In addition, over-expression of neutral amino acid transporter gene *OsAAP1* did not cause abnormal leaf development [35], which further indicated that Arg might be involved in the process of leaf development.

### 3.3. Abnormal Metabolic Pathway and Chloroplast Degradation in OsAAP3 OE Lines Might Cause Lesion Mimic and Leaf Senescence in Rice

Secondary metabolites are closely related to the PCD process. It has been reported that overexpression of lignin synthesis enzyme gene *OsAAE3* leads to an increase in H_2_O_2_ content, which triggers PCD induced by ROS [36]. Recently, a mutant *pir1* showed that the spontaneous lesions mimic phenotype is caused by PCD in the mutant leaves [32]. And KEGG analysis revealed that DEGs were most highly enriched in phenylpropanoid biosynthesis [32]. In this study, the expression levels in the flavonoids and phenolics pathway, which closely related to lignin metabolism, were changed in *OsAAP3* OE and Ri lines compared with WT (Figure S4). Then we speculate that the abnormal lignin biosynthesis in *OsAAP3* OE lines would trigger lesion mimic.

As important cellular organelles, chloroplasts are easily destroyed and degraded during the formation and persistence of lesion mimics [37]. Photosynthesis will be affected by the breakdown of chloroplasts and degradation of chlorophyll molecules [38,39]. Our results exhibited that the chlorophyll concentration decreased significantly in the leaves of the *OsAAP3* OE lines (Figure 1G,I). Further paraffin section and transmission electron section experiments observed that the chloroplast structure may have been disrupted in the leaves of the *OsAAP3* OE lines (Figure 3). Genes related to chloroplast degradation, chlorophyll synthesis, and photosynthesis are affected by lesion mimic and leaf senescence [40,41]. Therefore, our transcriptome analysis revealed that over-expression of *OsAAP3* leads to the abnormal gene expression of chloroplast development and photosynthesis pathway (Figure S5; Table S1). Besides, the abnormal expression of genes in transcriptome, such as LOC_Os08g44270 (*OsSAG12-2*) in lesion mimic pathway and LOC_Os03g30950 (*OsSAC3*) in leaf senescence pathway (Figure 4), caused rice leaf senescence and cell death in previous studies [42,43]. These results indicated that both abnormal metabolic pathway and chloroplast degradation of *OsAAP3* OE lines might cause lesion mimic and leaf senescence in rice.

In addition, various elements are remobilized to seeds after chloroplast degradation and leaf senescence, and macronutrients N, P and K are generally highly mobile in the phloem [44]. Our result showed that N and K decreased in the OE lines while the two elements increased in the Ri lines compared with WT, suggesting that accelerated chloroplast degradation and leaf senescence of *OsAAP3* OE lines might transfer more N and K to filling grains. Mg has not often been considered in studies on nutrient remobilization, and available results indicate a tendency of continued accumulation during leaf senescence [45]. Besides, Mn is the least phloem mobile among the micronutrients [44]. In this study, we indicated that the concentration of Mg and Mn increased in the OE lines, and the possible reason is that The Mg and Mn released from chloroplast degradation of *OsAAP3* OE lines transferred less to filling grains and remained more in leaves.

## 4. Materials and Methods

### 4.1. Plant Materials

All the transgenic materials of the *OsAAP3* OE lines, RNA interference (Ri) lines (as described in Lu et al. [17]), and wild-type (WT) zhonghua11 were grown in the rice experimental field of Guizhou University.

### 4.2. Physiological Index Analysis and Leaf Senescence Treatment

At the filling stage, flag leaves from the *OsAAP3* OE lines, Ri lines and WT were prepared for physiological index analysis. For amino acid Arg analysis, leaf blades were extracted with 10 mL 80% ethanol at 80 °C. A 1 mL aliquot of each sample was evaporated to remove the ethanol, re-dissolved in 1 mL 0.02 M HCl and subsequently analyzed using high-performance liquid chromatography [17]. Kits from Nanjing Jiancheng Technology Co., Ltd. (Nanjing, China) were used for the nitric oxide (NO), hydrogen peroxide (H_2_O_2_), proline (Pro), and malondialdehyde (MDA) measurements, as well as for detecting the enzyme activity of nitric oxide synthase (NOS), superoxide dismutase (SOD), catalase (CAT), and peroxidase (POD). For the leaf senescence treatment experiment, transgenic materials from the *OsAAP3* OE lines, Ri lines, and WT were reduced into fragments and immersed in an H_2_O_2_ solution for 4 days, after which the proportion of aging senescent leaves was counted. The chlorophyll concentration of the leaves was extracted with 80 % acetone in the dark for 24 h. The extract was measured by a spectrophotometer with light absorption values at 470, 645, and 663 nm.

### 4.3. Histochemistry Staining and Tissue Observation

For H_2_O_2_ detection, leaf samples were vacuum infiltrated with three cycles of 5 min each in ethanesulfonic acid (pH 6.5) containing 1 mg/mL 3, 3′-diaminobenzidine (DAB) and 10 mM 2-(N-morpholino), then were soaked in the above solution for 18 h in the dark. For superoxide determination, leaf samples were vacuum infiltrated three cycles of 5 min each in 10 mM potassium phosphate buffer (pH 7.8) containing 0.5 mg/mL nitro blue tetrazolium (NBT), then were soaked in the above solution for 16 h in the dark. Both the DAB and NBT staining reactions were stopped by 90% ethanol at 70 °C until chlorophyll was completely removed [46]. For membrane integrity detection, leaf samples were vacuum infiltrated with three cycles of 5 min in lactic acid-phenol-trypan blue solution (LPTB; 2.5 mg/mL trypan blue, 25% (*w*/*v*) lactic acid, 23% water-saturated phenol, and 25% glycerol in H_2_O) at 70 °C, then the samples in LPTB were heated in boiling water for 2 min. Following a 2 h cooled period, the LPTB solution was replaced with a chloral hydrate solution (25 g in 10 mL of H_2_O) for destaining [47].

### 4.4. Paraffin Section and Transmission Electron Section Analysis

For paraffin sections, leaf samples were held in a solution composed of 5 mL 4% paraformaldehyde and fixed in xylene for 20 min, and they were dehydrated by ethanol series. Then, 4 µm slices with Leica rotary microtome were stained in hematoxylin and eosin solution for 3 min, and observed under light microscope. For transmission electron section analysis, leaf samples were fixed in phosphate buffer solution (pH 7.2) with 2.5% glutaraldehyde at 4 °C for 4 h. The chloroplast ultrastructure of the samples was observed using a transmission electron microscope.

### 4.5. Transcriptome Analysis

Leaf samples were performed for RNA sequencing (RNA-seq) by Shanghai paisennuo Biotechnology Co., Ltd., China. The clean data were aligned to the rice genome reference sequence (Oryza_sativa. IRGSP-1.0) by HiSAT2 (v2.1.0) [48]. Transcripts were then assembled by stringtie (v2.0.1) [49] and processed using feature Counts to summarize the counting reads (subread-2.0.0) [50]. The intersections of differential genes analyzed by DESeq2 [false discovery rate (FDR) < 0.05 and fold change ≥ 2] were identified as differentially expressed genes (DEGs) [51].

### 4.6. Element Measurements

Leaf samples were dried at 80 °C for 3 d and then digested with nitric acid at 180 °C for 1 h with a MARS6 microwave. After the samples were diluted with deionized water, then measured by inductively coupled plasma mass spectrometry (ICP-MS, Agilent 7700 series; Agilent Technologies, Santa Clara, CA, USA).

### 4.7. Gene Expression Detection

Total RNA was extracted from leaves using TRIzol reagent (Nuoweizan, Nanjing, China). First-strand cDNA was synthesized using M-MLV reverse transcriptase (Takara, Shiga, Japan). from 2 μg of the total RNA. The first-strand cDNA was used as a template for RT-PCR after normalization using the rice Actin1 (AB047313). The RT-PCR was performed in a 10 μL reaction volume containing 1 μL of the cDNA solution, 1 μL gene-specific primers (10 μM), and 5 μL 2X SYBR PCR Mix (Nuoweizan, Nanjing, China) under the following conditions: 94 °C for 2 min (1 cycle), 94 °C for 30 s, 58 °C for 30 s, and 72 °C for 30 s (40 cycles), followed by 72 °C for 1 min (1 cycle). All the primers used in this study are listed in Table S2.

### 4.8. Statistical Analysis

For multiple comparisons, Duncan’s multiple range test was performed using SPSS software, indicating significant difference at *p* < 0.05.

## 5. Conclusions

This study indicated that over-expression (OE) of *OsAAP3* could lead to lesion mimic and leaf senescence in rice by regulating Arg transport and nitric oxide pathway. Furthermore, abnormal metabolic pathway and chloroplast degradation that were caused by oxidative burst from nitric oxide pathway in *OsAAP3* OE lines triggers lesion mimic in senescent leaves. This study will not only provide initial insights into the regulatory role of amino acid transport genes in rice growth and development but will also help to understand the factors regulating the leaf senescence.

## Supplementary Materials

The following are available online at https://www.mdpi.com/1422-0067/22/4/2181/s1, The following are available online at. Figure S1: Whole plant phenotype of the rice leaves with altered expression of *OsAAP3* grown in paddy field. Figure S2: Flag leaf phenotype of the overexpressing (OE) lines, RNA interference (Ri) lines, and wild-type (WT) under diaminobenzidine (DAB, A), nitroblue tetrazolium (NBT, B), and trypan blue (TB, C) staining. Figure S3: Proline (Pro) concentration of the OsAAP3 OE lines, Ri lines, and WT at the heading stage. Figure S4: Metabolic pathways covered by the transcriptional changes affecting the *OsAAP3* OE and Ri Lines. Figure S5: Differentially enriched genes in the nitrogen metabolism and photosynthesis pathways. Figure S6: RT-PCR analysis of genes involved in nitrogen metabolism and photosynthesis pathways. Figure S7: RT-PCR analysis of genes involved in lesion mimic and leaf senescence pathways. Table S1: The locus name and corresponding notes of differentially expressed genes among the OE line, Ri line, and WT in all related pathways. Table S2: List of the primers in this study.

## Abbreviations

Al	Aluminum
Arg	Arginine
CAT	Catalase
Cu	Copper
DAB	3, 3′-Diaminobenzidine
Fe	Iron
H_2_O_2_	Hydrogen peroxide
K	Potassium
Lys	Lysine
MDA	Malondialdehyde
Mg	Magnesium
Mn	Manganese
N	Nitrogen
Na	Sodium
NBT	Nitro blue tetrazolium
NO	Nitric oxide
NOS	Nitric oxide synthase
OE	Overexpressing
P	Phosphorus
PCD	Programmed cell death
POD	Peroxidase
Pro	Proline
Ri	RNA interference
RNA-seq	RNA sequencing
ROS	Reactive oxygen species
RT-PCR	Real-time quantitative PCR
SOD	Superoxide dismutase
TB	Trypan blue
WT	Wild-type

## References

[1] Wang S.A., Lei C.L., Wang J.L., Ma J., Tang S., Wang C.L., Zhao K.J., Tian P., Zhang H., Qi C.Y. (2017). SPL33, encoding an eEF1A-like protein, negatively regulates cell death and defense responses in rice. J. Exp. Bot..

[2] Xiao G., Zhou J., Lu X., Huang R., Zhang H. (2018). Excessive UDPG resulting from the mutation of UAP1 causes programmed cell death by triggering reactive oxygen species accumulation and caspase-like activity in rice. New Phytol..

[3] Ma J., Wang Y., Ma X., Meng L., Jing R., Wang F., Wang S., Cheng Z., Zhang X., Jiang L. (2019). Disruption of gene SPL35, encoding a novel CUE domain-containing protein, leads to cell death and enhanced disease response in rice. Plant Biotech. J..

[4] Ma X.M., Zhang Y.J., Tureckova V., Xue G.P., Fernie A.R., Mueller-Roeber B., Balazadeh S. (2018). The NAC transcription factor SlNAP2 regulates leaf senescence and fruit yield in tomato. Plant Physiol..

[5] Mhamdi A., Queval G., Chaouch S., Vanderauwera S., Van Breusegem F., Noctor G. (2010). Catalase function in plants: A focus on *Arabidopsis* mutants as stress-mimic models. J. Exp. Bot..

[6] Li Z., Zhang Y., Liu L., Liu Q., Bi Z., Yu N., Cheng S., Cao L. (2014). Fine mapping of the lesion mimic and early senescence 1 (*lmes1*) in rice (*Oryza sativa*). Plant Physiol. Biochem..

[7] Foyer C.H., Noctor G. (2005). Redox homeostasis and antioxidant signaling: A metabolic interface between stress perception and physiological responses. Plant Cell.

[8] Hu S., Yu Y., Chen Q., Mu G., Shen Z., Zheng L. (2017). OsMYB45 plays an important role in rice resistance to cadmium stress. Plant Sci..

[9] Yang Y., Xu J., Huang L., Leng Y., Dai L., Rao Y., Chen L., Wang Y., Tu Z., Hu J. (2016). PGL, encoding chlorophyllide a oxygenase 1, impacts leaf senescence and indirectly affects grain yield and quality in rice. J. Exp. Bot..

[10] Song G., Kwon C.T., Kim S.H., Shim Y., Lim C., Koh H.J., An G., Kang K., Paek N.C. (2019). The rice SPOTTED LEAF4 (SPL4) encodes a plant spastin that inhibits ROS accumulation in leaf development and functions in leaf senescence. Front. Plant Sci..

[11] Ke S., Liu S., Luanm X., Xiem X.M., Hsieh T.F., Zhang X.Q. (2019). Mutation in a putative glycosyltransferase like gene causes programmed cell death and early leaf senescence in rice. Rice.

[12] Klessig D.F., Durner J., Noad R., Navarre D.A., Wendehenne D., Kumar D., Zhou J.M., Shah J., Zhang S., Kachroo P. (2000). Nitric oxide and salicylic acid signaling in plant defense. Proc. Natl. Acad. Sci. USA.

[13] Guo F.Q., Crawford N.M. (2005). *Arabidopsis* Nitric Oxide Synthase 1 is targeted to mitochondria and protects against oxidative damage and dark-induced senescence. Plant Cell.

[14] Zago E., Morsa S., Dat J.F., Alard P., Ferrarini A., Inze D., Delledonne M., Van Breusegem F. (2006). Nitric oxide and hydrogen peroxide responsive gene regulation during cell death induction in tobacco. Plant Physiol..

[15] Lamattina L., Garcia-Mata C., Graziano M., Pagnussat G. (2003). Nitric oxide: The versatility of an extensive signal molecule. Annu. Rev. Plant Biol..

[16] Taylor M.R., Reinders A., Ward J.M. (2015). Transport function of rice amino acid permeases (AAPs). Plant Cell Physiol..

[17] Lu K., Wu B., Wang J., Zhu W., Nie H., Qian J., Huang W., Fang Z. (2018). Blocking amino acid transporter *OsAAP3* improves grain yield by promoting outgrowth buds and increasing tiller number in rice. Plant Biotech. J..

[18] Wu G., Sidney M., Morris J.R. (1998). Arginine metabolism: Nitric oxide and beyond. Biochem. J..

[19] Neill S.J., Desikan R., Hancock J.T. (2003). Nitric oxide signalling in plants. New Phytol..

[20] Lin A., Wang Y., Tang J., Xue P., Li C., Liu L., Hu B., Yang F., Loake J.G., Chu C. (2012). Nitric oxide and protein s-nitrosylation are integral to hydrogen peroxide-induced leaf cell death in rice. Plant Physiol..

[21] Del Río L.A. (2015). ROS and RNS in plant physiology: An overview. J. Exp. Bot..

[22] Qiu Z., Zhu L., He L., Chen D., Zeng D., Chen G., Hu J., Zhang G., Ren D., Dong G. (2019). DNA damage and reactive oxygen species cause cell death in the rice local lesions 1 mutant under high light and high temperature. New Phytol..

[23] Huang Z.A., Jiang D.A., Yang Y., Sun J.W., Jin S.H. (2004). Effects of nitrogen deficiency on gas exchange, chlorophyll fluorescence, and antioxidant enzymes in leaves of rice plants. Photosynthetica.

[24] Wang C.Y., Cheng S.H., Kao C.H. (1982). Senescence of rice leaves. 7. Proline accumulation in senescing excised leaves. Plant Physiol..

[25] Mondal W.A., Dey B.B., Choudhuri M.A. (1985). Proline accumulation as a reliable indicator of monocarpic senescence in rice cultivars. Experientia.

[26] Lee D., Lee G., Kim B., Jang S., Lee Y., Yu Y., Seo J., Kim S., Lee Y.H., Lee J. (2018). Identification of a spotted leaf sheath gene involved in early senescence and defense response in rice. Front. Plant Sci..

[27] Cui Y.J., Peng Y.L., Zhang Q., Xia S.S., Ruan B.P., Xu Q.K., Yu X.Q., Zhou T.T., Liu H., Zeng D.L. (2020). Disruption of EARLY LESION LEAF 1, encoding a cytochrome P450 monooxygenase, induces ROS accumulation and cell death in rice. Plant J..

[28] Bruggeman Q., Raynaud C., Benhamed M., Delarue M. (2015). To die or not to die? Lessons from lesion mimic mutants. Front. Plant Sci..

[29] Hung K.T., Kao C.H. (2004). Hydrogen peroxide is necessary for abscisic acid-induced senescence of rice leaves. J. Plant Physiol..

[30] Ge C., E Z., Pan J., Jiang H., Zhang X., Zeng D., Dong G., Hu J., Xue D. (2015). Map-based cloning of a spotted-leaf mutant gene *OsSL5* in *Japonica* rice. Plant Growth Regul..

[31] Balint-Kurti P. (2019). The plant hypersensitive response: Concepts, control and consequences. Mol. Plant Pathol..

[32] Chen X., Mei Q., Liang W., Sun J., Wang X., Zhou J., Wang J., Zhou Y., Zheng B., Yang Y. (2020). Gene mapping, genome-wide transcriptome analysis, and WGCNA reveals the molecular mechanism for triggering programmed cell death in rice mutant *pir1*. Plants.

[33] Manosalva P.M., Bruce M., Leach J.E. (2011). Rice 14-3-3 protein (GF14e) negatively affects cell death and disease resistance. Plant J..

[34] Wang J., Wu B., Lu K., Wei Q., Qian J., Chen Y., Fang Z. (2019). The amino acid permease 5 (OsAAP5) regulates tiller number and grain yield in rice. Plant Physiol..

[35] Ji Y., Huang W., Wu B., Fang Z., Wang X. (2020). The amino acid transporter AAP1 mediates growth and grain yield by regulating neutral amino acid uptake and reallocation in *Oryza sativa*. J. Exp. Bot..

[36] Liu H., Guo Z., Gu F., Ke S., Sun D., Dong S., Liu W., Huang M., Xiao W., Yang G. (2017). 4-Coumarate-CoA ligase-like gene *OsAAE3* negatively mediates the rice blast resistance, floret development and lignin biosynthesis. Front. Plant Sci..

[37] Matin M.N., Saief S.A., Rahman M.M., Lee D.H., Kang H., Lee D.S., Kang S.G. (2010). Comparative phenotypic and physiological characteristics of *spotted leaf 6* (*spl6*) and *brown leaf spot2* (*bl2*) lesion mimic mutants (LMM) in rice. Mol. Cells.

[38] Tamary E., Nevo R., Naveh L., Levin-Zaidman S., Kiss V., Savidor A., Levin Y., Eyal Y., Reich Z., Adam Z. (2019). Chlorophyll catabolism precedes changes in chloroplast structure and proteome during leaf senescence. Plant Direct.

[39] Lim P.O., Kim H.J., Nam H.G. (2007). Leaf senescence. Annu. Rev. Plant Biol..

[40] He L., Zhang S., Qiu Z., Zhao J., Nie W., Lin H., Zhu Z., Zeng D., Qian Q., Zhu L. (2018). FRUCTOKINASE-LIKE PROTEIN 1 interacts with TRXz to regulate chloroplast development in rice. J. Integr. Plant Biol..

[41] Li Z., Pan X., Guo X., Fan K., Lin W. (2019). Physiological and transcriptome analyses of early leaf senescence for *ospls1* mutant rice (*Oryza sativa* L.) during the grain-filling stage. Int. J. Mol. Sci..

[42] Zhao D., Oosterhuis D., Bednarz C. (2001). Influence of potassium deficiency on photosynthesis, chlorophyll content, and chloroplast ultrastructure of cotton Plants. Photosynthetica.

[43] Peng Y., Liao L., Liu S., Nie M., Li J., Zhang L., Ma J., Chen Z. (2019). Magnesium deficiency triggers SGR-mediated chlorophyll degradation for magnesium remobilization. Plant Physiol..

[44] Fischer A.M. (2007). Nutrient remobilization during leaf senescence. Annu. Plant Rev..

[45] Killingbeck K.T., Nooden L.D. (2004). Nutrient resorption. Plant Cell Death Processes.

[46] Qiao Y.L., Jiang W.Z., Lee J.H., Park B., Choi M.S., Piao R., Woo M.O., Roh J.H., Han L., Paek N.C. (2010). SPL28 encodes a clathrin-associated adaptor protein complex 1, medium subunit micro 1 (AP1M1) and is responsible for spotted leaf and early senescence in rice (*Oryza sativa*). New Phytol..

[47] Yin Z., Chen J., Zeng L., Goh M., Leung H., Khush G.S., Wang G.L. (2000). Characterizing rice lesion mimic mutants and identifying a mutant with broad-spectrum resistance to rice blast and bacterial blight. Mol. Plant Microbe Interact..

[48] Kim D., Langmead B., Salzberg S.L. (2015). HISAT: A fast spliced aligner with low memory requirements. Nat. Methods.

[49] Pertea M., Kim D., Pertea G.M., Leek J.T., Salzberg S.L. (2016). Transcript level expression analysis of RNA-seq experiments with HISAT, StringTie and Ballgown. Nat. Protoc..

[50] Liao Y., Smyth G.K., Shi W. (2014). FeatureCounts: An efficient general purpose program for assigning sequence reads to genomic features. Bioinformatics.

[51] Love M.I., Huber W., Anders S. (2014). Moderated estimation of fold change and dispersion for RNA-seq data with DESeq2. Genome Biol..

