# Supplementary material for: Altered Expression of OsAAP3 Influences Rice Lesion Mimic and Leaf Senescence by Regulating Arginine Transport and Nitric Oxide Pathway
Source: Int J Mol Sci. 2021 Feb 22;22(4):2181. doi: 10.3390/ijms22042181 (PMC7927093; doi:10.3390/ijms22042181)
Supplement: Supplementary file 1 [file ijms-22-02181-s001.pdf]

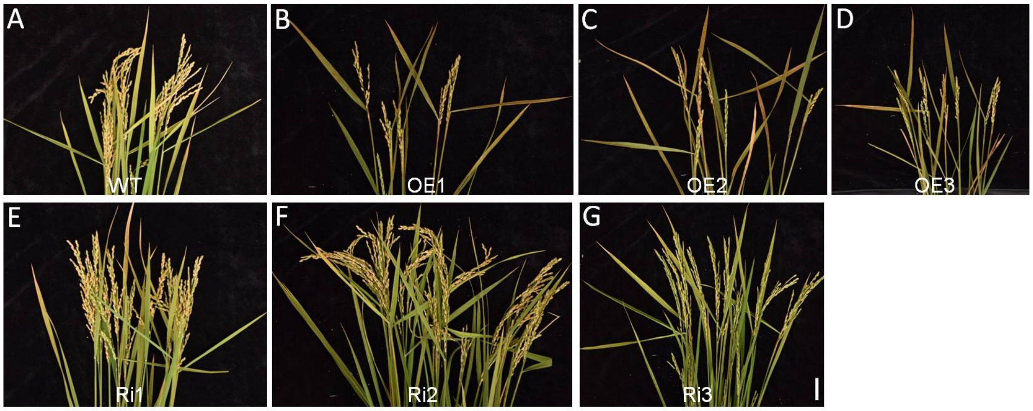

Figure S1. Whole plant phenotype of the rice leaves with altered expression of *OsAAP3* grown in paddy field.

WT (**A**) indicates wild-type ZH11, OE1-OE3 (**B-D**) indicate overexpressing lines and Ri1-Ri3 (**E-G**) indicate RNA interference lines. The flag leaves was taken at 120 days after sowing. Scale bar =3.0 cm in A-G.

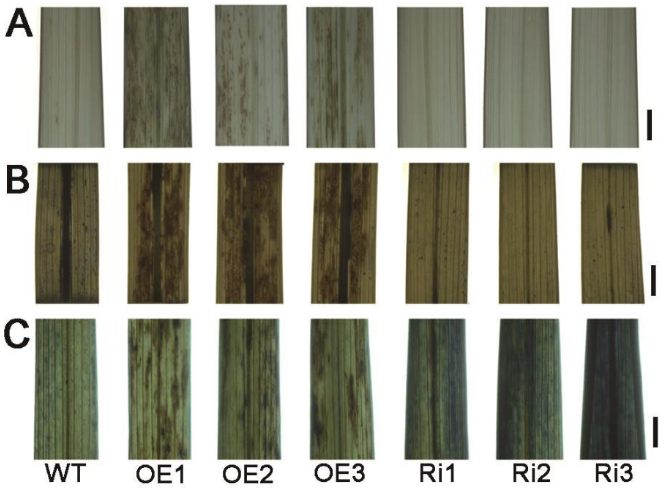

Figure S2. Flag leaf phenotype of the overexpressing (OE) lines, RNA interference (Ri) lines, and wild-type (WT) under diaminobenzidine (DAB, **A**), nitroblue tetrazolium (NBT, **B**), and trypan blue (TB, **C**) staining.

The flag leaves was taken at 95 days after sowing. Scale bar =0.5 cm in A-C.

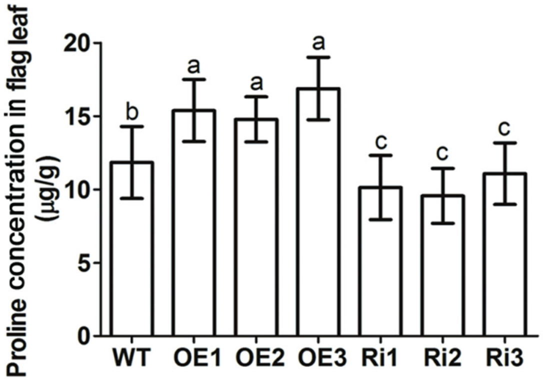

Figure S3. Proline (Pro) concentration of the *OsAAP3* OE lines, Ri lines, and WT at the heading stage. The flag leaves was taken at 110 days after sowing. The letters above the error bars are ranked by the Duncan test at $p<0.05$. Values are means ± standard deviation (n=5).

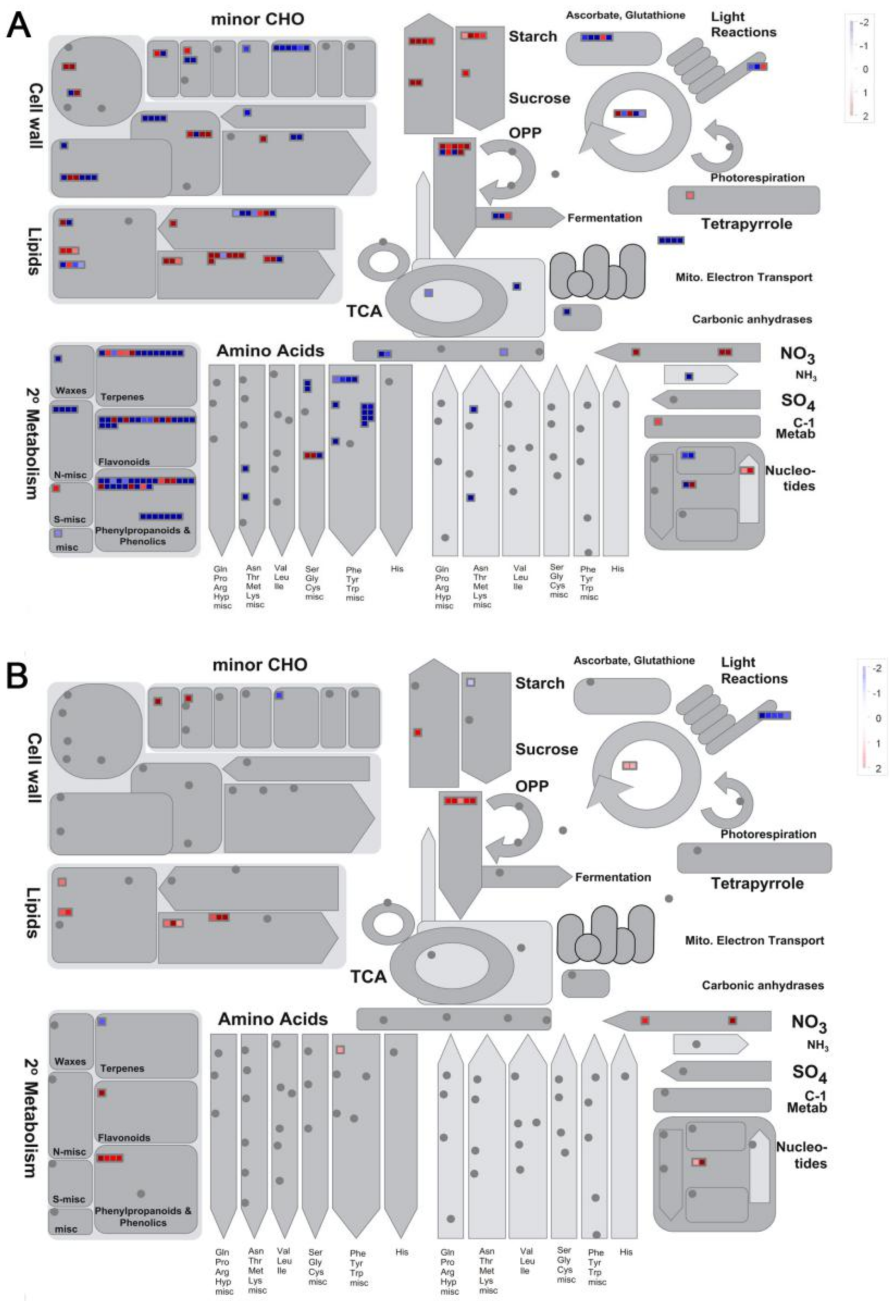

Figure S4. Metabolic pathways covered by the transcriptional changes affecting the *OsAAP3* OE and Ri Lines.

Overview of expression changes in the metabolic pathways in leaves of the overexpressing (OE) (**A**) and RNA interference (Ri) lines (**B**) using MapMan software. Only the genes showing a significant ($P<0.05$) change in expression between OE and WT and between Ri and WT are represented and were attributed to the respective bins by MapMan. Genes whose expression increased or decreased are shown by an increasingly intense blue and red color, respectively. The flag leaves was taken at 110 days after sowing.

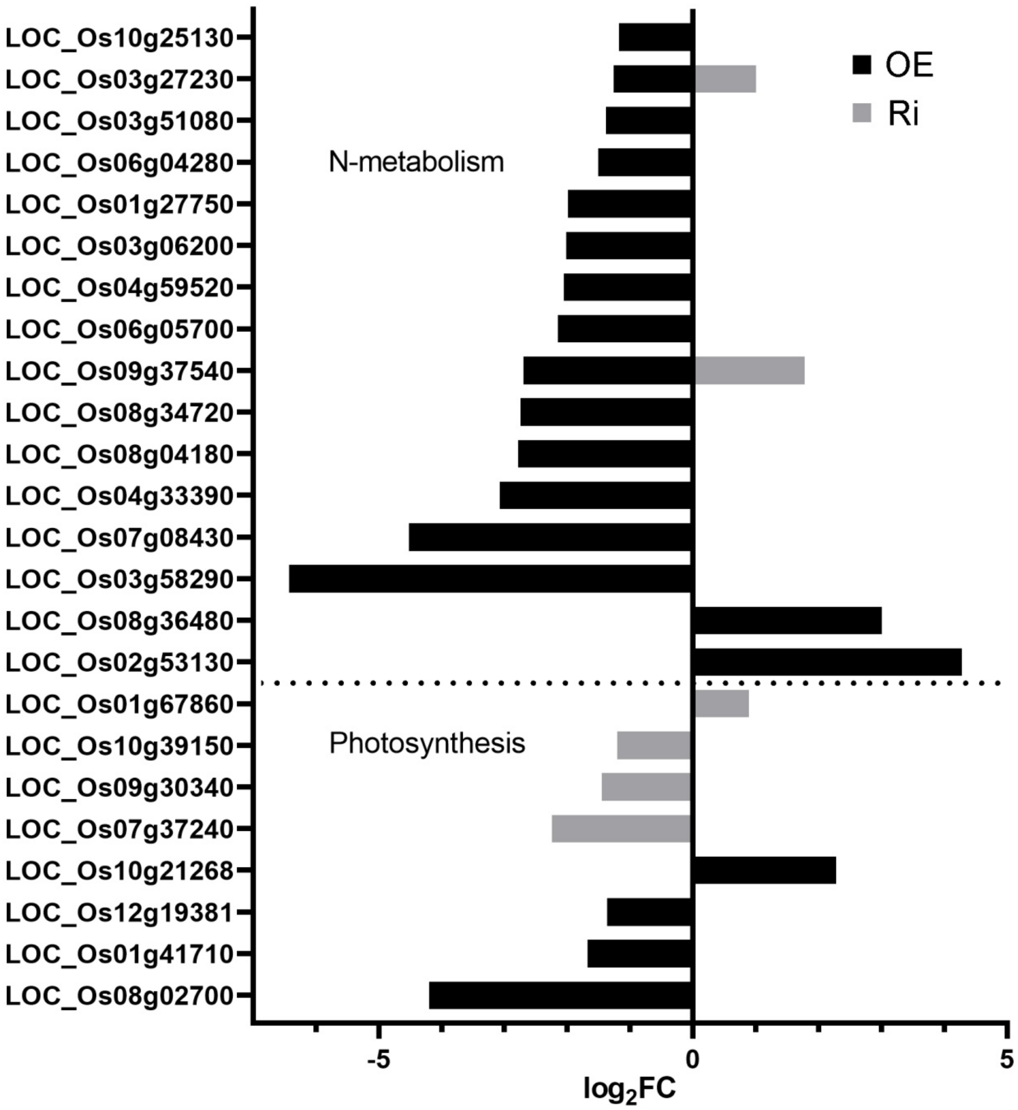

Figure S5. Differentially enriched genes in the nitrogen metabolism and photosynthesis pathways. The abscissa is log2 Fold Change, and the ordinate is Locus Name of gene.

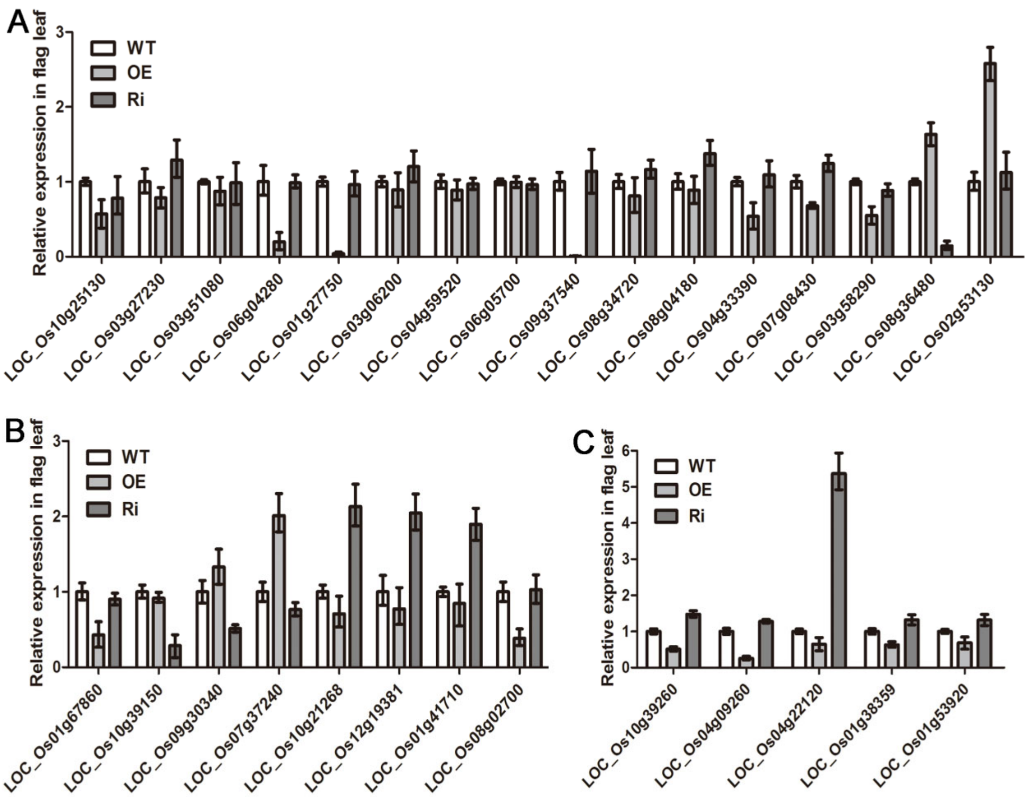

Figure S6. Quantitative reverse transcription PCR analysis of genes involved in nitrogen metabolism and photosynthesis pathways.

(**A**) Genes in nitrogen metabolism pathway from Figure S5. (**B**) Genes in photosynthesis metabolism pathway from Figure S5. (**C**) Differentially expressed genes among the OE line, Ri line, and WT from Figure 5B. The transcript levels of the first genes were defined as "1". Data are means ± standard deviation (n=3). WT indicates the wild-type, OE indicates the overexpression line, and Ri indicates the RNA interference line.

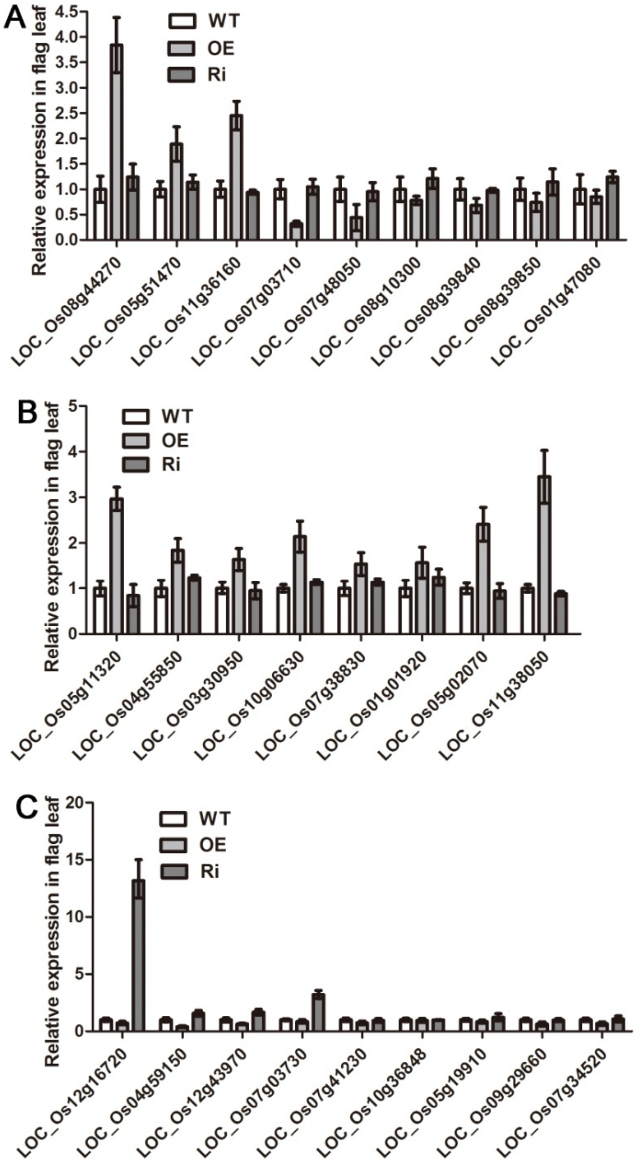

Figure S7. RT-PCR analysis of genes involved in lesion mimic and leaf senescence pathways.
(**A**) Genes in lesion mimic pathway from Figure 4. (**B**) Increased expression genes of *OsAAP3* OE lines in leaf senescence pathway from Figure 4. (**C**) Decreased expression genes of *OsAAP3* OE lines in leaf senescence pathway from Figure 4. The transcript levels of the first genes were defined as "1". Data are means ± standard deviation (n=3). WT indicates the wild-type, OE indicates the overexpression line, and Ri indicates the RNA interference line.

Table S1. The locus name and corresponding notes of differentially expressed genes among the OE line, Ri line, and WT in all related pathways.

Locus Name	Gene Product Name	Classification
LOC_Os08g44270	vignain precursor	Lesion mimic
LOC_Os05g51470	2-aminoethanethiol dioxygenase	
LOC_Os11g36160	receptor-like protein kinase 2 precursor	
LOC_Os07g03710	SCP-like extracellular protein	

LOC_Os07g48050	peroxidase precursor	
LOC_Os08g10300	SHR5-receptor-like kinase	
LOC_Os08g39840	lipoxygenase, chloroplast precursor	
LOC_Os08g39850	lipoxygenase, chloroplast precursor	
LOC_Os01g47080	pyruvate kinase	
LOC_Os05g11320	metallothionein-like protein 3B	Leaf senescence
LOC_Os04g55850	nuclease PA3	
LOC_Os03g30950	acyl-desaturase, chloroplast precursor	
LOC_Os10g06630	peptidyl-prolyl cis-trans isomerase	
LOC_Os07g38830	hydrolase, alpha/beta fold family domain containing protein	
LOC_Os01g01920	HD domain containing protein 2	
LOC_Os05g02070	expressed protein	
LOC_Os11g38050	phosphoesterase family protein	
LOC_Os12g16720	cytochrome P450 71A1	
LOC_Os04g59150	peroxidase precursor	
LOC_Os12g43970	hydrolase, alpha/beta fold family domain containing protein	
LOC_Os07g03730	SCP-like extracellular protein	
LOC_Os07g41230	esterase	
LOC_Os10g36848	cytochrome P450	
LOC_Os05g19910	transferase family protein	
LOC_Os09g29660	white-brown complex homolog protein 11	
LOC_Os07g34520	isocitrate lyase	
LOC_Os10g25130	aminotransferase	Nitrogen metabolism
LOC_Os03g27230	phospho-2-dehydro-3-deoxyheptonate aldolase	
LOC_Os03g51080	glutamate decarboxylase	
LOC_Os06g04280	3-phosphoshikimate 1-carboxyvinyltransferase	
LOC_Os01g27750	bifunctional 3-dehydroquinate dehydratase/shikimate dehydrogenase	
LOC_Os03g06200	aminotransferase	
LOC_Os04g59520	anthranilate phosphoribosyltransferase	
LOC_Os06g05700	cysteine synthase	
LOC_Os09g37540	uncharacterized protein PA4923	
LOC_Os08g34720	D-3-phosphoglycerate dehydrogenase	
LOC_Os08g04180	tryptophan synthase beta chain 1	
LOC_Os04g33390	prephenate dehydratase domain containing protein	
LOC_Os07g08430	indole-3-glycerol phosphate lyase	
LOC_Os03g58290	indole-3-glycerol phosphate lyase	
LOC_Os08g36480	nitrate reductase	
LOC_Os02g53130	nitrate reductase	
LOC_Os01g67860	fructose-bisphospate aldolase isozyme	Photosynthesis

LOC_Os10g39150	unspecified	
LOC_Os09g30340	photosystem I reaction center subunit, chloroplast precursor	
LOC_Os07g37240	chlorophyll A-B binding protein	
LOC_Os10g21268	ribulose bisphosphate carboxylase large chain precursor	
LOC_Os12g19381	ribulose bisphosphate carboxylase small chain, chloroplast precursor	
LOC_Os01g41710	chlorophyll A-B binding protein	
LOC_Os08g02700	fructose-bisphospate aldolase isozyme	
LOC_Os10g39260	aspartic proteinase nepenthesin	Differentially expressed genes among the OE line, Ri line, and WT in Figure 5B.
LOC_Os04g09260	transferase family protein	
LOC_Os07g03730	SCP-like extracellular protein	
LOC_Os04g22120	protein kinase	
LOC_Os09g37540	uncharacterized protein PA4923	
LOC_Os01g38359	peptidyl-prolyl cis-trans isomerase, FKBP-type	
LOC_Os12g16720	cytochrome P450 71A1	
LOC_Os01g53920	receptor-like protein kinase 5 precursor	
LOC_Os05g35290	phenylalanine ammonia-lyase	
LOC_Os05g15770	glycosyl hydrolase	
LOC_Os05g05680	1-aminocyclopropane-1-carboxylate oxidase	
LOC_Os03g27230	phospho-2-dehydro-3-deoxyheptonate aldolase, chloroplast precursor	
LOC_Os07g42960	phospho-2-dehydro-3-deoxyheptonate aldolase, chloroplast precursor	

Table S2. List of the primers in this study.

Gene	Forward sequence (5' - 3')	Reverse sequence (5' - 3')
Actin	CGGTGTCATGGTCGGAAT	GCTCGTTGTAGAAGGTGT
LOC_Os07g34520	CGGACAGGTTCCGGCTCACCAA	CGTAGACGGTGTCGAGGTGCTTCG
LOC_Os09g29660	AACCCACCGTGCCGAGGTGGAA	GCTGATCTCCAGCGACGGCT
LOC_Os05g19910	ACGTGTTCCGCCGCCACAAGA	GTTCACGTCGTCGAGGCTGCA
LOC_Os10g36848	TCTGAGCCTGGCGATCGTCGT	AGGTGCATCAGGCCGCCGTACT
LOC_Os07g41230	ATGGGGTCGCTGATGTCCTGCATC	AACGAGCTCCTCTGCCCCAGCA
LOC_Os07g03730	CCAGCTTGCTAGTAGCTGCAGTG	TACTTCCCGCCGGAGTCCGAGT
LOC_Os12g43970	CATCCATGGCTTCCCGGAGCT	CAGAGATGCCACGCCACCTGAGCT
LOC_Os04g59150	GTCCAAGCTGGGTATGGTTGTGCT	ACGTGCCACCTCACGATGCCCT
LOC_Os12g16720	TGGAGCTCACCATGGCGTCGA	CATGAGGTGGAGGTGCCCGATCA
LOC_Os11g38050	GAGAACCGCTCCTTCGACCACATG	ACCTGCTCGTAGATGGCCTGGA
LOC_Os05g02070	TGCAGATGCAAGATGTTCCCTGA	CGACGATAGATTCAGTTGCAGGA
LOC_Os01g01920	TCTCGCGCGATTCTCGGAGGAGGA	CAGCCGTAGTAGTGGGGTTGCTT

LOC_Os07g38830	ACGTCCTCCAGTGGCCACCA	ATGGAGGAGGCCTCGACACGCA
LOC_Os10g06630	AGGGTGTTCCTTGACATCGCCA	GTGCACAGCCGCCGGAAGTT
LOC_Os03g30950	AGCAACAGCCTCGTCAGCCT	TCCACCCACGCGTTCAGCGA
LOC_Os04g55850	AGATCGCCGAGGGTTTCCTG	TGGCAATCCCGGGCGTAGTT
LOC_Os05g11320	CTGCGCTGACAAGAGCCAGTGCGT	AGCAGCCGGTGGTGCACTTG
LOC_Os01g47080	CGCAAGACAAAGATCGTGTGCA	TGGTTGAGGCAAATCTCCACTCC
LOC_Os08g39850	ATGCTGCGTCCCCAGCTCAA	GCACCTCACAACAACTCGCTGC
LOC_Os08g39840	CCATCTAGCCACACGACGACGA	CGCACACAACAACAACCCGACT
LOC_Os08g10300	ATCGCGTGCTCATGGGTTGCAGT	GCAGTCGCACTTGATGCCGGGGTT
LOC_Os07g48050	AGCTGTCGGCGACGTTCTACGA	TGCGCCTTGATGCTGTCGATGACG
LOC_Os07g03710	TGGCGAGTTCGTCGAGCAGGTT	GAGTCCGAGTGCTCCAGCTTGCAG
LOC_Os11g36160	TGGCGAGATCACTCGCGTTG	CCATGTGCAGTGCTGGCCGT
LOC_Os05g51470	AAGGTGGCGGAACCCAAGGACC	AAGACCTCCCTGCTGGTGTCGAAG
LOC_Os08g44270	TGTGGGAGCTGTACGAGCGGT	CGGAACTCGTCGGCGGTCATGT
LOC_Os08g02700	GCGTGCTACAAGGCGCTGAG	CACCTGGTTCATGGCGTTCA
LOC_Os01g41710	GCGGTCCAGGTGGTGCTCAT	GCCCAGGCGTTGTTGTTGA
LOC_Os12g19381	CGATCCAAGTGGGTGCCT	CGATGATGCGAACAAATGC
LOC_Os10g21268	GGCAGCATTCCGAGTAAC	AACCCTCTTCAAATAGGTCTAA
LOC_Os07g37240	TGGTGGATGGGTCGTCGTAC	TGCAGCCGCTCCTTCTTCT
LOC_Os09g30340	GGGGCGGTTCGTGTTCTTC	GTTTGGGTTGTATCCGTTGCTG
LOC_Os10g39150	TGCAAGAGGCTGGCGAGGAA	CCATGAGACCAGGAACGATAGG
LOC_Os01g67860	TGCCTGCCATTGTCTTCC	CGAGCACAGCATCACCCT
LOC_Os02g53130	ACAACTGCTGGTTCAGGGTGA	TGGTGTACTGCGTGGTGGC
LOC_Os08g36480	GGAGGACACGACGGAGATGC	GCGAGACGGCGAACTTGA
LOC_Os03g58290	GCCATTAAGAGCAACCTAGAGC	TGATATGCCAAAGCCAACAC
LOC_Os07g08430	TTGGGCGTGCCTTACTCT	TGGACACCGGCTTCTTTT
LOC_Os04g33390	TGCGGATCTCGGATCTGTCG	CGTAGTTGCGGTGGATGCTG
LOC_Os08g04180	TCAACAATGCTGTCGCTCAA	CCCTCACCTCTGCTCCAAG
LOC_Os08g34720	GCGGTAATGGGCTTTGGC	TTCGTCGTTGAAGACCTTGGAT
LOC_Os09g37540	GGCGGGTTTGCGTGTTCT	TTGCCCATCAGAGTGGTAGGT
LOC_Os06g05700	TGGGTTTAGGATTGGTGC	CCTGATTTGCTGGATTTGA
LOC_Os04g59520	GGTGGTCGTCAAGGACAAGGA	GCGAGGCTGTCAAGGGAGA
LOC_Os03g06200	TACAAGACGCACGCGGACAA	GCGAGGGTGAACGGCACAT
LOC_Os01g27750	TGGTCATGCCCTGAGATT	CGCTGACAACTTTAATCCC
LOC_Os06g04280	AACTTGGTGCGGATGTCG	TGTACTTCTGCCCTCCCTTA
LOC_Os03g51080	GAAACAGGCTGGGACACG	AGTTGAGGGTGAAGGTGGG
LOC_Os03g27230	GAGAAGAGCCGCATCCCT	GTTAGCCCAGCAGCAGACA
LOC_Os10g25130	GAGGGACTTGTTCTTCTGG	TCACTGACGCCACTTTGT
LOC_Os10g39260	TGGCAACGCTGCTGGTGCTGTG	TGCACTGCGTCCAGACGAGCT
LOC_Os04g09260	TGACACGCCTGGGCATGTCGT	ACGTCCTCCAGGGTGCAGTT
LOC_Os04g22120	CACCTTCAGCTATGACGCGGA	TGAAGCTCGCGGCGAAGCTG
LOC_Os01g38359	CAACAGGAGGTTCTTCGTGTGGG	ATCCTCGGGTTTGGAGCACTTGG

	T	
LOC_Os01g53920	ACGTTCCCGACGAGCGTGTA	CAGCTTCTGCAATCGGGACAAC
